# The Evolving Scope of *PLoS Neglected Tropical Diseases*

**DOI:** 10.1371/journal.pntd.0000379

**Published:** 2009-02-24

**Authors:** Peter J. Hotez, Gavin Yamey

**Affiliations:** 1 Department of Microbiology, Immunology, and Tropical Medicine, The George Washington University, Washington, D.C., United States of America; 2 Sabin Vaccine Institute, Washington, D.C., United States of America; 3 Public Library of Science, San Francisco, California, United States of America

*PLoS Neglected Tropical Diseases* is an open-access community journal that serves the needs of a small but active and robust community of neglected tropical disease (NTD) scientists, clinicians, and public health and policy experts. As stewards of that community, our editorial staff has very much molded the journal according to what we have learned from you in terms of mission, priorities, and scope. In the eighteen months since the launch of *PLoS Neglected Tropical Diseases*, some of our most interesting editorial discussions and queries from authors are in regards to the journal's scope, particularly the specific conditions defined as NTDs (http://www.plosntds.org/static/scope.action).

What has emerged from these discussions is a journal focused on a fairly specific group of important infections endemic in developing countries with seven common features [1],[2]: (1) the NTDs occur in the setting of poverty where they are the most prevalent infections afflicting “the bottom billion,” i.e., people living on less than US$1 per day [3]; (2) they are chronic conditions—people can harbor their NTDs for years or decades; (3) they generally disable, rather than kill; (4) they are frequently disfiguring and stigmatizing [4]; (5) they are not typically emerging in character; instead, the NTDs have plagued humankind for centuries and many are described in ancient texts [5]; (6) they are not just diseases *of* poverty, but they also *promote* poverty because of their effects on child development, cognition, and education, adult agricultural worker productivity, and pregnancy outcome [3]; and (7) the NTDs are a critical component of the “other diseases” mentioned in the sixth Millennium Development Goal (“Combat HIV/AIDS, malaria, and other diseases”) [1],[2],[6].

## Helminths

There are certain diseases that easily meet these criteria. A good example are the helminth infections, especially the most common ones, such as hookworm infection, ascariasis, trichuriasis, strongyloidiasis, schistosomiasis, and lymphatic filariasis—each affecting more than 100 million people in sub-Saharan Africa or tropical regions of Asia and the Americas. We have included many of the less common helminthiases in our scope, including food-borne trematode infections, onchocerciasis, loiasis and other filarial infections, cysticercosis and other cestodiases, and other intestinal nematode infections. We also include helminth infections that are not exclusively tropical, such as enterobiasis, toxocariasis, and trichinellosis. In North America (and elsewhere in temperate regions) helminth infections such as ascariasis and strongyloidiasis are still considered diseases that primarily affect the poor, and they have been designated by some as “neglected infections of poverty” [7],[8]. In summary, we would consider almost any human helminth infections as an NTD and thus within the scope of the journal.

## Protozoa

Similarly, most of the protozoan infections, including Chagas disease, leishmaniasis, human African trypanosomiasis, and many of the intestinal protozoan infections, are also considered NTDs. Conspicuous by its absence on the protozoan infection list is malaria. Certainly, no one would question the devastating global health impact of this disease, nor its predilection to affect the poor. Moreover, as with the NTDs, several investigators, including Jeffrey Sachs and others, have documented in some depth the poverty-promoting impact of malaria [9],[10]. We have chosen to omit malaria from our list of NTDs for three reasons. First, there is a comparatively large community of investigators working on malaria (in contrast to the smaller NTD research community). Second, while we acknowledge shortfalls in funding [11], there have been important infusions of funding and heightened advocacy for malaria control in recent times, including through the US President's Malaria Initiative, The Global Fund to Fight AIDS, Tuberculosis, and Malaria, Roll Back Malaria, and several malaria advocacy groups, including Malaria No More (http://www.malarianomore.org/). Third, malaria researchers already have a very wide range of open-access venues for their work, including the other six PLoS journals, all of which have published studies on malaria, as well as the *Malaria Journal*, published by BioMed Central (http://www.malariajournal.com/).

However, we continue to invite and publish malaria papers that examine malaria and NTD co-infection or co-endemicity, such as a recent study we published examining whether helminth infection in children affects malaria susceptibility [12] and another that surveyed trachoma and malaria together in Ethiopia [13]. In 2007, Ric Price and colleagues made a cogent argument for considering vivax malaria as a neglected disease and we largely agree with their comments [14]. But after some lengthy editorial discussions, we have decided for the moment to hold off on opening the door completely to papers on vivax malaria both because of the availability of other open-access journals that have malaria in their scope, as outlined above, and because of our concerns that the number of submissions on this topic would overwhelm the papers we are currently defining as NTDs. Having said that, we want *PLoS Neglected Tropical Diseases* to be a “living” journal that reflects the needs of the community and our scope will thus continue to evolve over time. Therefore, on a case-by-case basis we will consider papers on vivax malaria and related topics.

## Bacteria and Fungi

When it comes to the bacterial infections, chronic infections such as leprosy, Buruli ulcer, and trachoma clearly qualify as NTDs. Similarly, leptospirosis, relapsing fever, and the treponematoses are important neglected bacterial diseases. We are not generally considering papers on tuberculosis for publication for much the same reason that we refrain from considering malaria papers, i.e., there is a sizeable group of comparatively well-funded experts in the field of tuberculosis and the disease falls in the scope of the other six PLoS journals. However, we are currently considering aspects of bovine tuberculosis that pertain to health in developing countries. Since the launch of PLoS *Neglected Tropical Diseases*, we have had several lively editorial discussions about the important enteric bacterial infections, such as cholera, salmonellosis, and shigellosis. After talking to several experts in the field, including Richard Guerrant and Gerald Keusch, we are now welcoming papers on these topics, particularly as they relate to disease in developing countries. We continue to consider papers on tropical fungal infections, as well as some selected non-infectious NTDs, such as podoconiosis.

## Viruses

We are not considering papers on HIV/AIDS unless they pertain to NTD co-infections—an example of a paper we have published on HIV–NTD co-infection was the systematic review by Judd Walson and Grace John-Stewart that examined whether treating helminth infection affects the prognosis of patients with HIV-1 [15]. Among the other viral infections, we now welcome papers on arboviral infections and have taken measures to add experts in this area on our editorial board. In addition, we recognize the importance of rabies as an NTD, as well as some of the viral hemorrhagic fevers. Our journal continues to publish papers on the insect vectors that transmit NTD pathogens, as well as papers on intermediate hosts such as snails.

We consider *PLoS Neglected Tropical Diseases* a work in progress that operates in an iterative manner in order to best serve the needs of the community. It is our hope that the journal will continue to attract new researchers to the field and, by its heavy representation of editorial staff from endemic countries, continue to build scientific and public health capacity in developing countries. We are working particularly hard to reach out to authors in low-income settings (see http://www.plosntds.org/static/developing.action), for example by providing additional editorial support to authors whose first language is not English. We are especially sensitive to supporting women in the sciences, medicine, and public health and this is also reflected in the composition of our editorial board. We further expect that papers published in the Magazine section will help to foster a cadre of NTD policy experts. From the number and quality of submissions received to date we feel that we are meeting those needs. At the same time, we very much want to hear from you about how we might continue to fine tune and improve the journal in order to ensure that open access continually enhances this important and vital pursuit. Please send us your feedback by adding your annotations to this article or starting a discussion thread using our innovative online tools.
